# Impact of Carbon Diffusion Induced Stress on the Properties of Diamond/GaN Heterojunctions

**DOI:** 10.3390/nano16040241

**Published:** 2026-02-12

**Authors:** Haolun Sun, Mei Wu, Peng Xu, Chao Yuan, Ling Yang, Hao Lu, Bin Hou, Meng Zhang, Xiaohua Ma, Yue Hao

**Affiliations:** 1 National Engineering Research Center of Wide Band-Gap Semiconductor, Faculty of Integrated Circuit, Xidian University, Xi’an 710126, China; haolun_sun@stu.xidian.edu.cn (H.S.);; 2 School of Integrated Circuits, Wuhan University, Wuhan 430072, China

**Keywords:** polycrystalline diamond, carbon diffusion, thermal boundary resistance, stress modulation, AlGaN/GaN heterostructures

## Abstract

Integrating diamond with GaN provides an effective pathway to mitigate self-heating. However, the thermal boundary resistance (TBR) remains a persistent bottleneck for further heat dissipation. While carbon (C) diffusion into the SiNx interlayer is known to reduce TBR, the associated stress evolution and its impact on device performance remain underexplored. In this work, the synergistic regulation of heat transport and electrical performance induced by C diffusion was systematically investigated. Transmission electron microscopy (TEM) was employed to characterize the interfacial microstructure and the influence of C diffusion on the interface. To further assess the resulting impact on heat dissipation, transient thermoreflectance was utilized to precisely quantify the thermal transport within the heterostructures. Classical molecular dynamics (MD) simulations were then performed to analyze the underlying physical mechanisms, revealing that intensifying C diffusion increases the phonon density of states overlap and effectively reduces the TBR. Furthermore, the intrinsic stress was quantified through geometric phase analysis (GPA) based on TEM images, demonstrating that the stress induced during the diffusion process propagates to the AlGaN/GaN heterostructure. Crucially, this stress modulation enhances the piezoelectric polarization by approximately 32%, resulting in a 5% increase in the two-dimensional electron gas (2DEG) sheet density. These findings provide a comprehensive strategy for optimizing the thermal management and mechanical reliability of high-power GaN devices.

## 1. Introduction

GaN-based high electron mobility transistors (HEMTs) have emerged as the backbone of next-generation high-power radio frequency (RF) and microwave applications due to their high breakdown voltage and superior electron saturation velocity [1,2,3,4,5]. However, their performance potential is severely restricted by the self-heating effect. As power density increases, heat accumulation within the active region leads to a sharp decline in two-dimensional electron gas (2DEG) mobility, causing a marked degradation in both device characteristics and reliability [6,7,8]. Therefore, efficient near-junction thermal management has become a critical challenge to fully realize the potential of GaN technology.

To address this, integrating a high thermal conductivity (TC) diamond film on the top of GaN devices is considered the most effective strategy [9,10,11,12,13]. A SiNx dielectric layer is typically employed to protect the GaN surface from H_2_ etching damage before diamond growth. Although the introduction of this amorphous layer solves the integration problem of the diamond/GaN heterostructure, its poor TC and interfacial phonon matching inevitably result in a higher thermal boundary resistance (TBR) at the diamond/GaN interface, which becomes a significant obstacle to the effective upward heat dissipation of GaN devices [14,15,16,17,18]. To overcome this limitation, previous studies have indicated that the specific growth conditions of diamond, particularly the high-power density and chamber pressure, promote the diffusion of C atoms into the SiNx interlayer. This diffusion process facilitates the formation of localized SiC nanocrystals within the amorphous layer. The resulting structural transformation effectively bridges the phonon mismatch and enhances the TC of the dielectric layer, achieving an extremely low TBR [19,20,21,22]. To deeply comprehend the internal mechanisms driving these properties, current research increasingly adopts a synergy of experiment and simulation. Specifically, calculations based on Density Functional Theory (DFT) have been employed to elucidate the energetics of carbon (C) diffusion and its interactions with defects or dangling bonds, thereby providing theoretical guidance for understanding the subsequent thermal/electronic transport [23]. Moreover, molecular dynamics (MD) simulations serve as a bridge to macroscopic properties, utilizing interatomic potentials to model the phonon density of states (PDOS) at the mixed interface, thereby capturing the dynamic impact of C-induced crystallization [24]. Despite extensive experimental validation confirming the structural evolution caused by C diffusion, a comprehensive understanding of the governing internal mechanisms and their influence on coupled electro-thermal properties is still lacking. On the one hand, the specific influence of C-induced stress evolution on the TBR necessitates in-depth theoretical study [25,26]. On the other hand, the propagation of stress into the AlGaN/GaN heterojunction and its subsequent modulation of electrical properties through the piezoelectric effect are not yet fully understood [27,28,29]. Consequently, a combined strategy of experiment and simulation is indispensable to systematically elucidate the impact of C diffusion on the coupled electro-thermal performance of AlGaN/GaN heterostructures.

In this work, we systematically investigated the stress variation induced by varying degrees of C diffusion and its dual impact on the TBR and the electrical characteristics of the AlGaN/GaN heterojunction. MD simulations were employed to verify the influence of C diffusion-induced stress on the TBR. Furthermore, geometric phase analysis (GPA) based on transmission electron microscopy (TEM) was utilized to quantitatively characterize the stress propagation into the AlGaN/GaN heterostructure.

## 2. Materials and Methods

To elucidate the impact of C diffusion induced stress on the coupled electro-thermal performance, samples with identical epitaxial structures were prepared on SiC substrates. The heterostructure consisted of a 1.4 μm GaN buffer, a 0.8 nm AlN interlayer, and a 25 nm Al_0.25_Ga_0.75_N barrier. A ~40 nm SiNx passivation layer was deposited through metalorganic chemical vapor deposition (MOCVD). During diamond growth, a double-stage process was employed to balance the competing effects of etching and deposition by adjusting the growth parameters. In the nucleation stage, the extent of C diffusion was controlled by tuning the plasma power density, chamber pressure, and growth temperature, thereby enabling the fabrication of samples with varying dielectric layer thicknesses and C diffusion levels.

To measure the thermal properties of the diamond/SiNx/GaN interfaces, the transient thermoreflectance (TTR) method was utilized [30,31,32]. The TTR system uses a 355 nm pulse laser as the pump beam. Pulse laser irradiates the sample and produces heating in the surface, which penetrates into the interior of the sample and passes through each material. A continuous wave (CW) 532 nm laser was used as the probe beam, focused on the sample, and co-axial aligned with the pump beam. Au/Ti metal transducers (80 nm/5 nm) are deposited on the surface of the diamond. The metal transducers generate quantifiable thermal signals from the surface by having a precise linear relationship between reflectance change (∆R/R) and temperature change (∆T). The temperature change signal carried by the reflected probe beam is detected by a photodetector (with a bandwidth of 400 MHz) and displayed on a digital oscilloscope (with a bandwidth of 500 MHz).

To complement the experimental investigation and provide atomistic insights into the role of C diffusion, molecular dynamics (MD) simulations were conducted using LAMMPS (Large-scale Atomic/Molecular Massively Parallel Simulator) to investigate the diffusion behavior of C atoms within amorphous SiNx layers. The amorphous SiNx layer was generated using a melt-quench protocol [33,34,35]. The simulation domain employed periodic boundary conditions along the x- and y-directions, while fixed boundary conditions were maintained in the z-direction to ensure proper heat flow analysis. The Lennard–Jones (LJ) potential was implemented to characterize the atomic pair interactions between localized SiC crystalline and amorphous SiNx. The specific potential function for the SiC is described in the references [36]. To evaluate vibrational matching at the interface, the *PDOS* was calculated for various configurations [37,38]. The AlGaN layer in contact with the interlayer is described using the Stillinger–Weber (SW) potential, with dimensions commensurate with the actual film size [39].

## 3. Results and Discussion

**Figure 1:**
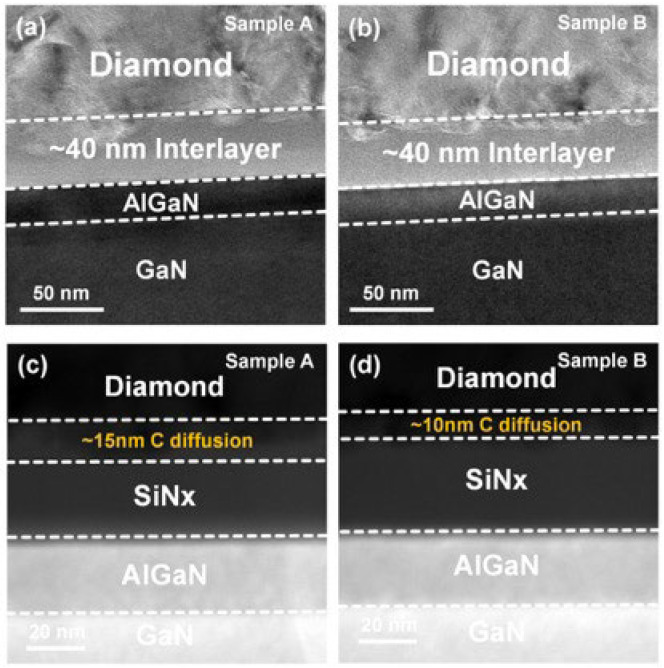
Cross-sectional TEM microstructure of the diamond/GaN interfaces of (**a**) sample A and (**b**) sample B; corresponding STEM images showing interfacial regions of (**c**) samples A and (**d**) sample B.

The microstructural characteristics of the fabricated heterostructures were examined using TEM, as shown in Figure 1. Both samples A and B exhibited a continuous AlGaN/GaN heterostructure capped with an approximately 40 nm SiNx dielectric layer, which effectively protects the GaN surface from H_2_ etching during diamond growth. Despite the identical initial SiNx thickness, varying plasma power densities, chamber pressures, and growth temperatures during diamond synthesis resulted in different degrees of C diffusion. The scanning transmission electron microscopy (STEM) image in Figure 1c,d further confirms the layer thicknesses and reveals that the C diffusion exhibits an island-like distribution, consistent with the island nucleation mechanism of diamond growth. In sample A, a 15 nm thick C diffusion layer was observed together with a remaining 25 nm amorphous SiNx layer. In contrast, sample B showed a reduced C incorporation with a 10 nm diffusion layer and a 30 nm SiNx layer [40]. These observations establish a clear correlation between the extent of C diffusion and the remaining SiNx thickness, providing a structural foundation for subsequent TBR analysis and polarization effect investigations.

**Figure 2:**
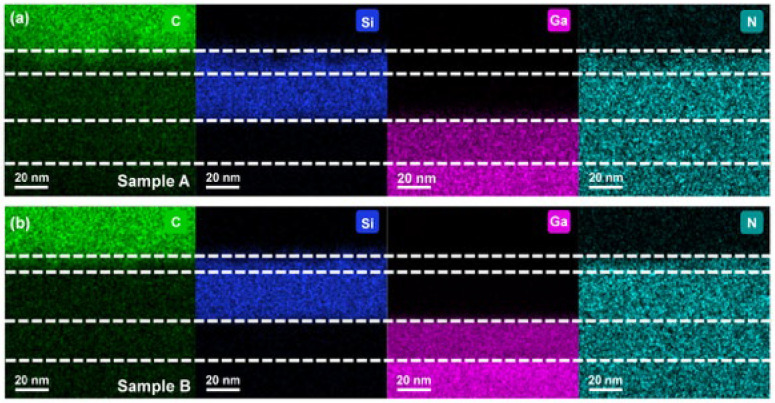
Elemental EDS mapping of (**a**) sample A and (**b**) sample B, showing the distributions of C, Si, Ga, and N.

To further verify the nature of the diffusion layer observed in the TEM images, energy-dispersive X-ray spectroscopy (EDS) mapping was performed for C, silicon (Si), gallium (Ga), and nitrogen (N) elements, as shown in Figure 2. The elemental distributions clearly reveal that both samples exhibited C diffusion into the SiNx dielectric layer, leading to the formation of a SiC layer, while no penetration into the GaN region was detected [19,22]. In sample A, the extent of C diffusion was more pronounced, resulting in a thicker SiC layer and a correspondingly thinner amorphous SiNx layer. In contrast, sample B showed a similar transformation but with reduced C incorporation, leading to a relatively thicker SiNx layer. The thicknesses of the diffusion and dielectric layers determined from EDS mapping were consistent with those obtained from TEM observations, thereby confirming that the contrast variations observed in the TEM images originated from C diffusion.

### 3.1. Impact of Stress on Heterojunction Thermal Transport

**Figure 3:**
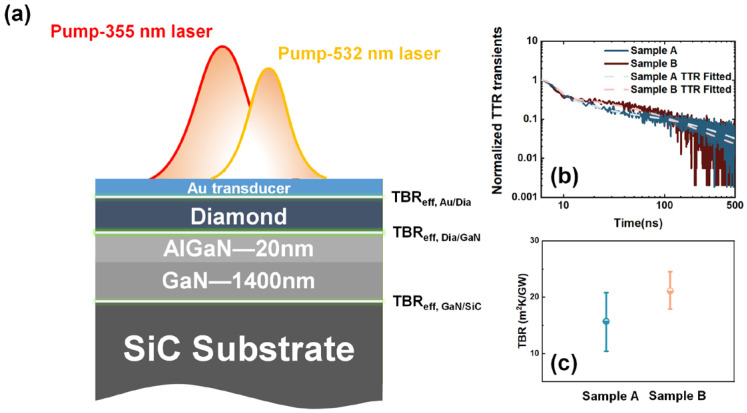
(**a**) Schematic of diamond/SiNx/GaN multilayer composite structure used for TTR measurements. (**b**) TTR transients measured on sample A and sample B with best fitting curves overlaid with the measured transients. (**c**) Comparative fitting results and TBR error analysis for Samples A and B.

To evaluate the thermal properties of the diamond/SiNx/GaN interfaces, the TTR results are presented in Figure 3. The measured TTR signal is fitted using an analytical heat conduction model (by Global Optimization in MATLAB R2021a) to extract the unknown thermophysical properties of the measured samples [41]. As illustrated in Figure 3a, the Ti layer is incorporated into the effective TBR of the Au/diamond interface (TBR_eff, Au/Dia_). Similarly, the AlGaN barrier and GaN buffer are treated as a single GaN layer, while the diamond/SiNx, SiNx, and SiNx/GaN interfaces are collectively represented as the effective TBR of the diamond/GaN interface (TBR_eff, Dia/GaN_). Figure 3b shows the measured TTR transients for representative samples A and B, with the best fitting curves overlaid with the measured transients, where the TTR transients of samples A and B highlight clear differences in heat dissipation behavior. Furthermore, Figure 3c presents the comparative fitting results and error analysis for samples A and B. Sample A, characterized by a thicker C diffusion layer and thinner amorphous SiNx dielectric, exhibited a lower TBR compared to sample B. In contrast, the reduced C incorporation and relatively thicker SiNx layer in sample B resulted in a higher TBR.

**Figure 4:**
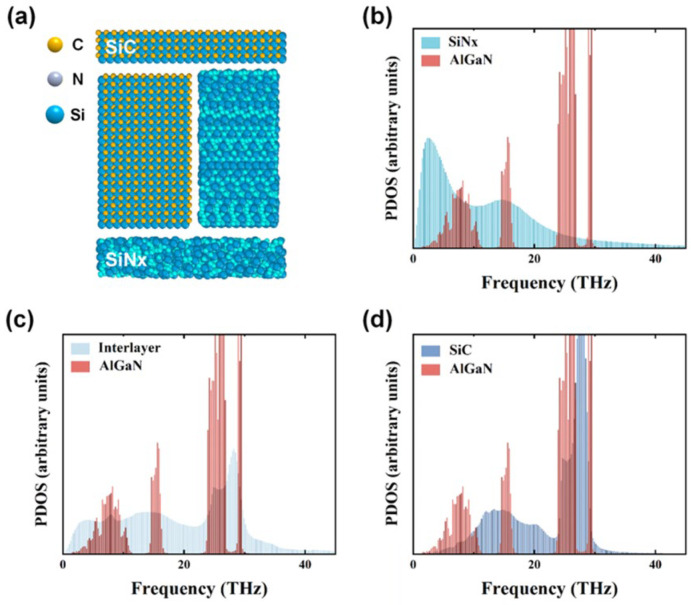
(**a**) Schematic of the MD model structure. PDOS comparison of the (**b**) SiNx layer, (**c**) interlayer, and (**d**) SiC layer with AlGaN in the diamond/GaN heterostructures.

Further structural analysis revealed that increased C diffusion promoted partial crystallization within the amorphous SiNx layer while simultaneously reducing its residual thickness, both of which contributed to a lower TBR. To investigate this mechanism, MD simulations were conducted using LAMMPS with the modeling framework illustrated in Figure 4a. The model domain consisted of a top SiC layer, a middle diffusion layer combining amorphous SiNx and SiC, and a bottom amorphous SiNx layer. As shown in Figure 4b–d, with increasing C diffusion, the PDOS of the interlayer gradually shifted toward that of SiC, leading to a significantly larger overlap with AlGaN vibrations. This enhanced phonon coupling provides a microscopic explanation for the experimentally observed reduction in thermal boundary resistance. Previous studies have demonstrated that increased C diffusion enhances the thermal conductivity of the diffusion layer, thereby reducing the overall TBR. Furthermore, the simulation indicates that the crystallization of SiC alters the internal layer structure, enlarging the overlap of the PDOS between the diffusion layer and the AlGaN region, which in turn lowers the interfacial resistance at the lower interface. The underlying reason may be attributed to the modification of phonon velocity with increasing C diffusion. According to the DMM, such variation in phonon velocity enhances the transmission coefficient between the two materials, reduces phonon scattering probability, and consequently decreases the interfacial thermal resistance [42,43].

### 3.2. Impact of Stress on AlGaN/GaN Polarization Effects

In addition to its influence on interfacial thermal transport, the stress induced by C diffusion significantly affects the polarization effects of the AlGaN/GaN heterostructure. During the high-temperature growth of diamond, C diffuses into amorphous SiNx and causes local crystallization, which changes the internal structure and creates stress. As the C diffusion becomes more intense and the SiNx layer becomes thinner, the impact of stress on the interface becomes more significant, which eventually causes stronger lattice strain in the AlGaN layer. The piezoelectric polarization, which originates from lattice mismatch and strain at the heterointerface, directly governs the formation and density of the 2DEG. As the lattice of AlGaN changes, the polarization within the heterojunction is also modified [44].

**Figure 5:**
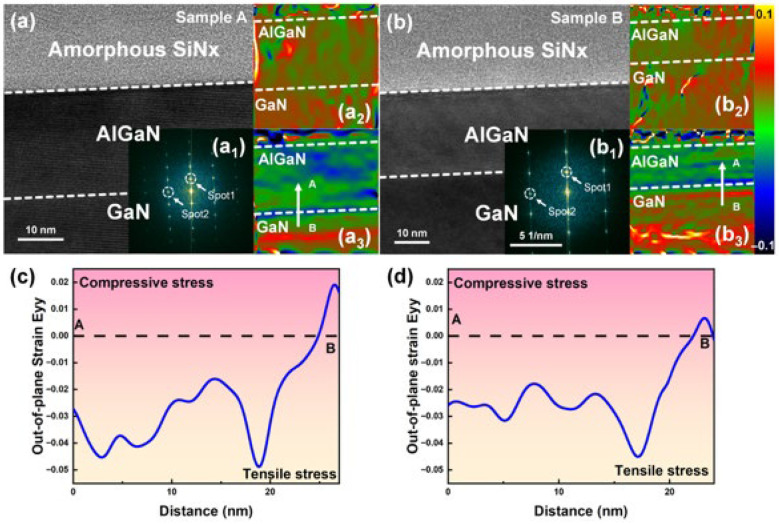
TEM images of AlGaN/GaN heterostructures for (**a**) sample A and (**b**) sample B. Selected FFT patterns used for GPA analysis of (**a_1_**) sample A and (**b_1_**) sample B, and the corresponding strain maps along the xx and yy directions of (**a_2_**,**a_3_**) sample A and (**b_2_**,**b_3_**) sample B. Line-scan profiles extracted from the strain maps of (**c**) sample A and (**d**) sample B.

To quantitatively evaluate this stress distribution, geometric phase analysis (GPA) was performed on high-resolution TEM images, as shown in Figure 5. Both samples exhibited comparable AlGaN/GaN layer thicknesses, providing a consistent basis for strain comparison. The corresponding fast Fourier transform (FFT) patterns of the heterointerfaces are presented in Figure 5(a_1_,b_1_), where the strongest conjugate diffraction spots in the horizontal and vertical directions were selected. These reflections were used to reconstruct the stress field distributions along both orientations, with the unstrained GaN buffer serving as the zero-strain reference. During the calculation, strain profiles were integrated perpendicular to the interface, and average strain values were extracted from the bulk region of the AlGaN barrier to eliminate interfacial peaks [28,29].

The in-plane (ε_xx_) and out-of-plane strain (ε_yy_) distribution maps for both samples are displayed in Figure 5(a_2_,a_3_,b_2_,b_3_). For the ε_xx_ maps, no distinct boundary or discontinuity was observed across the heterointerface. In contrast, the ε_yy_ maps exhibited a sharp discontinuity due to lattice mismatch and Poisson effect [45]. A localized strain peak was consistently observed at the immediate interface, reflecting atomic-scale distortion. As shown in Figure 5c,d, line-scan profiles along the A–B direction further confirmed that sample A, with stronger C diffusion and a thinner SiNx dielectric, introduced enhanced tensile stress into the AlGaN region. In contrast, sample B showed weaker stress modulation. By averaging the bulk strain values, the compressive strain was determined to increase from –0.025 in sample B to –0.033 in sample A, corresponding to a 32% enhancement in stress within the AlGaN barrier. According to piezoelectric theory, this mechanical gain leads to a proportional increase in the piezoelectric polarization component. Considering the dilution effect of spontaneous polarization in GaN-based heterostructures, the 32% stress enhancement is theoretically translated into a net increase of approximately 5% in the 2DEG sheet density [46,47].

## 4. Conclusions

In conclusion, C diffusion-induced stress modulation exerts a dual positive effect on AlGaN/GaN heterostructures. By controlling the C diffusion depth to approximately 15 nm within the 40 nm SiNx interlayer, the TBR is reduced due to better phonon transmission. At the same time, this C-induced stress effectively changes the lattice strain in the AlGaN layer, leading to a 32% increase in piezoelectric polarization and a 5% enhancement in the 2DEG sheet density. These quantitative results suggest that increasing the C diffusion intensity while keeping the AlGaN/GaN interface intact is a practical way to improve both the thermal and electrical performance of GaN devices.

## Data Availability

The datasets presented in this article are not readily available because the data are part of an ongoing study. Requests to access the datasets should be directed to corresponding author.
